# A dual-regulation inducible switch system for microRNA detection and cell type-specific gene activation

**DOI:** 10.7150/thno.84111

**Published:** 2023-04-23

**Authors:** Wen-Jie Shu, Kyungwoo Lee, Zhe Ma, Xiaojie Tian, Jong Seung Kim, Fu Wang

**Affiliations:** 1 School of Basic Medical Sciences, Xi'an Jiaotong University, Xi'an 710061, China.; 2 Department of Chemistry, Korea University, Seoul 02841, Korea.; 3 Engineering Research Center of Molecular and Neuro Imaging, Ministry of Education, School of Life Science and Technology, Xidian University, Xi'an 710071, China.; 4 Xianyang Key Laboratory of Molecular Imaging and Drug Synthesis, School of Pharmacy, Shaanxi Institute of International Trade & Commerce, Xianyang 712046, Shaanxi, China.

**Keywords:** microRNAs, cell type-specific, gene regulation, p21, Bax

## Abstract

**Rationale:** MicroRNAs (miRNAs) play key roles in multiple biological processes, many of which exhibit distinct cell type-specific expression patterns. A miRNA-inducible expression system can be adapted as a signal-on reporter for detecting miRNA activity or as a cell type-specific gene activation tool. However, due to the inhibitory properties of miRNAs on gene expression, few miRNA-inducible expression systems are available, and the available systems are only transcriptional or post-transcriptional regulatory system with obvious leaky expression.

**Methods:** To address this limitation, a miRNA-inducible expression system that can tightly control target gene expression is desirable. Here, by taking advantage of an enhanced LacI repression system and the translational repressor L7Ae, a miRNA-inducible dual transcriptional-translational switch system was designed called the miR-ON-D system. Luciferase activity assay, western blotting, CCK-8 assay and flow cytometry analysis were performed to characterize and validate this system.

**Results:** The results demonstrated that leakage expression was strongly suppressed in the miR-ON-D system. It was also validated that the miR-ON-D system could be used to detect exogenous and endogenous miRNAs in mammalian cells. Moreover, it was shown that the miR-ON-D system could be triggered by cell type-specific miRNAs to regulate the expression of biologically relevant proteins (e.g., p21 and Bax) to achieve cell type-specific reprogramming.

**Conclusion:** This study established a tight miRNA-inducible expression switch system for miRNA detection and cell type-specific gene activation.

## Introduction

MicroRNAs (miRNAs) are a class of small noncoding RNAs that can bind to specific mRNA targets and induce their degradation and/or translational inhibition 1. Increasing evidence has shown that miRNAs play important regulatory roles in a variety of biological processes, such as differentiation, development, and tumorigenesis 2, 3. In particular, many miRNAs exhibit distinct cell type-specific expression patterns, and their expression may change dynamically during physical and pathological processes 4. Additionally, miRNAs are considered biomarkers for a variety of cancer types 5. Accurate detection of miRNA expression is beneficial for revealing the functions of miRNAs and evaluating their cellular states. However, traditional miRNA detection approaches usually require cell lysis, such as northern blotting, microarrays, and quantitative reverse transcription PCR, which cannot reflect the dynamic changes in miRNA expression in living cells 6. Thus, a reporter for detecting and visualizing miRNA expression in living cells is urgently required. A miRNA-inducible expression system can be adapted as a signal-on reporter for miRNA activity, which is thought to provide a more reliable evaluation of miRNA expression than a signal-off reporter 6-8. Moreover, based on the cell type-specific expression properties of miRNAs, a miRNA-inducible expression system can be adapted as a cell type-specific gene activation tool for cell type-specific reprogramming. However, owing to the inhibitory properties of miRNAs on gene expression, very few miRNA-inducible positive systems are available.

A common strategy for constructing miRNA-inducible expression systems is to construct a negative-feedback gene circuit 8, 9. In these circuit systems, after transfecting circuit plasmids into cells, the expression of the target miRNA inhibits the repressor, thereby activating the expression of the gene of interest (GOI). Interestingly, Wang *et al*. developed a synthetic toehold switch that can be activated by miRNAs and demonstrated its capability for detecting and visualizing miRNA expression in mammalian cells 10. However, to the best of our knowledge, most of the reported miRNA-inducible expression systems are single-level (transcriptional or post-transcriptional) regulatory systems. Among the synthetic biology systems, single-level regulatory systems usually have some leakage expression, wherein the expression of a GOI is observed in its uninduced state, which often leads to the poor performance of genetic circuits 11-13. As for miRNA-inducible expression systems, leaky gene expression seriously affects their in vivo applications 14. For example, once the system is used to regulate the expression of fluorescent proteins, such as GFP, for the visual detection of miRNA, leaky GFP expression results in a high background fluorescence signal. In addition, if the system is used to study the function of a GOI, leaky expression results in the inability to determine whether a phenotype is the result of a threshold response to changes in gene expression. Moreover, the cell type-specific gene activation function of the miRNA-inducible expression system may be impaired due to leaky gene expression. One notable example is that when a cell type-specific miRNA is used to activate toxic gene expression to kill specific cell types, low levels of leaky expression may also lead to non-target cell death, as even faint toxic gene expression may kill host cells 15. Therefore, a tight miRNA-inducible expression system with low leakage is highly desirable.

**Scheme 1:**
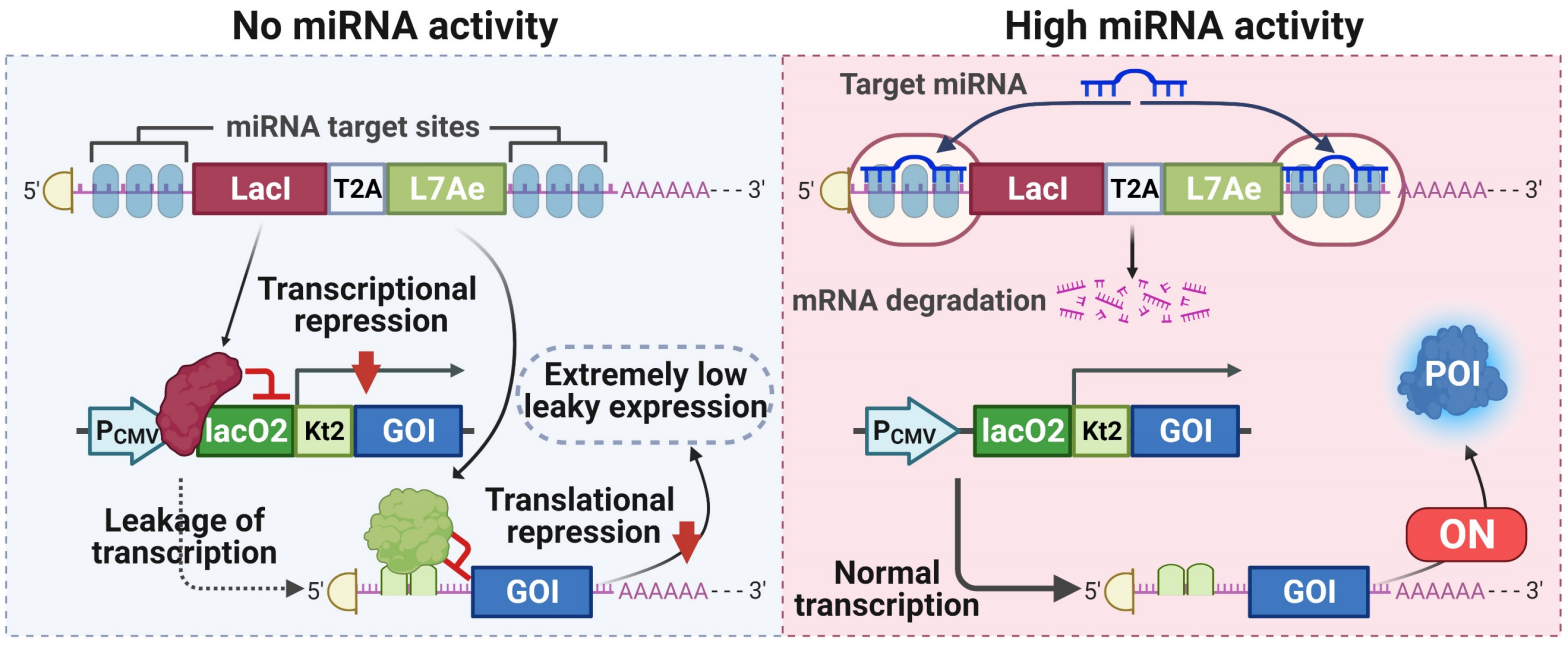
** A tight miRNA-inducible switch by coupling transcriptional and translational regulation.** In off-target cells lacking the target miRNA, the expression of LacI protein inhibits the transcription of the gene of interest (GOI), and the expression of L7Ae protein mediates translational suppression of the leaked transcripts of the GOI, ultimately minimizing the leakage expression. On the contrary, in on-target cells expressing the target miRNA at high levels, the expression of both LacI and L7Ae protein is inhibited, thereby releasing transcriptional and translational inhibition of the GOI.

To this end, in this study, an enhanced LacI repression system, which produces stronger transcriptional repression than the wild-type lac system 16, was taken advantage of to construct a miRNA-inducible transcriptional regulatory system with low leakage expression. A miRNA-inducible translational regulatory system was assembled using the translational repressor L7Ae. Considering that both the transcriptional and translational regulation system may still show obvious leaky expression, the above transcriptional and translational regulatory elements were coupled to develop a dual transcriptional-translational control system (miR-ON-D) that was activated by miRNA (Scheme 1). Leakage expression was strongly suppressed in the miR-ON-D system. It was also shown that the miR-ON-D system could be used to detect exogenous and endogenous miRNAs in mammalian cells. Moreover, the results demonstrated that the miR-ON-D system could effectively inhibit proliferation or induce apoptosis of target cells by activating p21 or Bax expression. This study establishes a tight miRNA-inducible dual-regulation switch system for miRNA detection and cell type-specific gene activation and reprogramming.

## Methods

### Plasmid construction

All the required DNA fragments (Supplementary Table 1) were synthesized by Tsingke Biotechnology Co., Ltd. and then cloned into the pCl-neo vector. Plasmid DNA was prepared from *Escherichia coli* using the EndoFree Mini Plasmid Kit II (TIANGEN BIOTECH (BEIJING) Co., Ltd., Beijing, China). All plasmid DNA was sequenced and identified using appropriate primers (Tsingke Biotechnology Co., Ltd.).

### Cell culture

All mammalian cells were cultured in DMEM with high glucose (Thermo Fisher Scientific) supplemented with 10% FBS and 1% penicillin/streptomycin solution. Cells were maintained at 37°C and 5% CO_2_. For C2C12 cell myogenic differentiation, when cell confluence reached 80-90%, C2C12 cells were transferred into differentiation medium containing 2% horse serum in DMEM.

### Cell transfection

All plasmid transfections were performed using the Hieff Trans^TM^ Liposomal Transfection Reagent (YEASEN, Cat:40802ES03). The day before transfection, cells were trypsinized and seeded at a density of 1 × 10^5^ cells per well in a 24-well plate with 0.5 mL of medium. Transfections were performed when the cells reached 90-95% confluence. DNA plasmids were diluted in 50 μL serum-free medium (Opti-MEM). The transfection reagent was diluted with 50 μL of Opti-MEM. After 5 min of incubation at 25°C, the diluted DNA and diluted transfection reagents (total volume of 100 µL) were mixed gently and incubated at room temperature for 20 min and then added to the cells.

Lipofectamine 2000 (Thermo Fisher Scientific) transfection reagent was used for plasmid DNA and miRNA mimic co-transfection. Plasmid DNA and miRNA mimics were diluted in Opti-MEM and mixed with diluted Lipofectamine 2000. After incubation for 10 min at room temperature, the mixed samples were added to each well. After 5 h of transfection, the culture medium was replaced with fresh medium. The miRNA mimics used in this study were obtained from GenePharma (Supplementary Table 2).

### Luciferase activity assay

After 24 h of transfection, the cells were washed with PBS and lysis buffer was added to lyse the cells. After incubation on ice for 5 min to fully lyse the cells, they were centrifuged at 12,000 rpm for 1 min and the supernatant was removed. Then, 20 μL of lysate from each sample was added to each well of a 96-well plate, and 100 μL of firefly luciferase (Fluc) detection reagent (YEASEN, Cat:11401ES76) was added. After mixing, the luminescence intensity of each sample was measured using a multimode reader.

### Western blotting

The cells were transfected into a 12-well plate. After 24 h of transfection, cells were harvested and lysed with RIPA buffer. The supernatant was carefully transferred to a new centrifuge tube after centrifuged at 12,000 × *g* for 5 min. Protein loading buffer was added to the supernatant. The sample was boiled for 10 min and centrifuged at 12,000 rpm for 1 min. The protein samples were separated on SDS-polyacrylamide gels and transferred to PVDF membranes. Next, the PVDF membranes were blocked with 5% milk and probed with primary antibodies, including ACTB (Epizyme, LF201), HA (Proteintech, 51064-2-AP), or FLAG (Proteintech, 20543-1-AP). After incubation with the primary antibody, the membranes were washed three times with TBST for 10 min (replicated three times). The membranes were further incubated with horseradish peroxidase-conjugated secondary antibody. Finally, chemiluminescence instruments were used to visualize the protein bands.

### CCK-8 assay

Cell proliferation was measured using the Cell Counting Kit-8 (CCK-8) (YEASEN, Cat:40203ES76). Experiments were performed according to the manufacturer's instructions. Briefly, 5 × 10^3^ cells were seeded in each well of a 96-well plate. At 0, 24, 48, or 72 h, 10 μL of CCK-8 reagent was added to each well of the 96-well plate, and the cells were incubated for 2 h. The absorbance was measured at 450 nm using a multimode reader.

### Flow cytometry analysis

Cell samples were prepared using an Annexin V-FITC/PI Apoptosis Detection Kit (YEASEN, Cat:40302ES50). Briefly, six-well plate cells were digested with EDTA-free 0.1% trypsin. The cells were washed twice with ice-cold PBS and centrifuged for 5 min at 500 × *g* at 4°C. The supernatant was discarded and the cell pellets were resuspended in 1× binding buffer. The cell suspension (100 μL) was added to 5 μL of Annexin V-FITC solution and 10 μL of PI solution and mixed gently. After 15 min of reaction at room temperature in the dark, 400 μL of 1× binding buffer was added. The samples were analyzed by flow cytometry within 1 h.

### Statistical analysis

All the data are representative of three independent experiments. All quantitative data were analyzed using Student's* t*-test and are presented as the mean ± standard deviation. Except where indicated otherwise, a *P* value of less than 0.01, 0.001, or 0.0001 was considered statistically significant and marked as *, **, or ***, respectively. 'ns' indicates 'not significant.'

## Results and Discussion

### Design and characterization of a miRNA-inducible transcriptional regulation system

**Figure 1:**
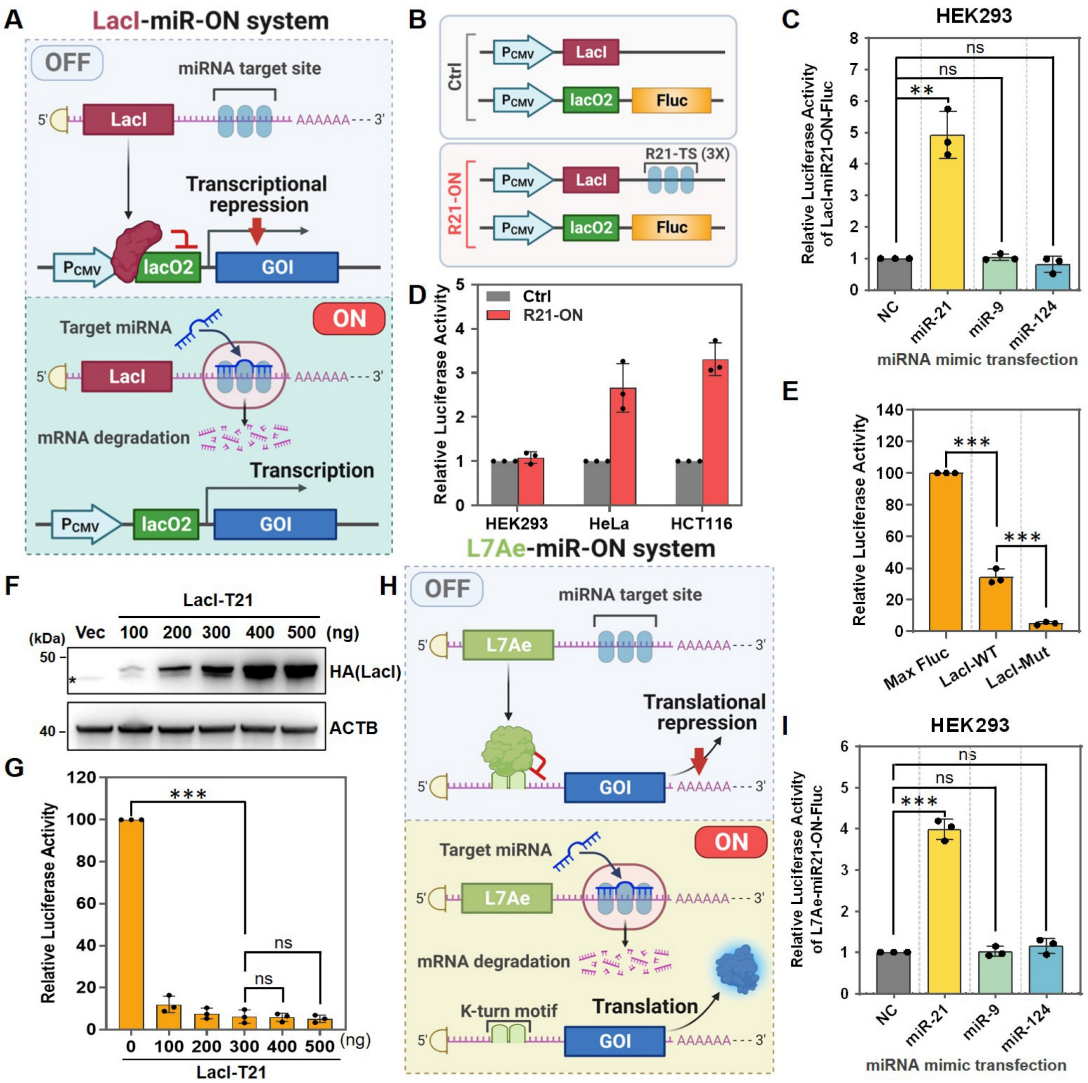
** Design and characterization of the miRNA-inducible transcriptional or translational regulation system.** (**A**) Scheme for the LacI-miR-ON system that contains two plasmids: pLacI and pLacO. For pLacI, three copies of miRNA target sequences were incorporated into the 3'-UTR of *LacI,* whereas two copies of the lac operator (lacO2) were incorporated into the 5'-UTR of the gene of interest to obtain pLacO. (**B**) Scheme for LacI-miR21-ON-Fluc system plasmids (R21-ON) or control system plasmids (Ctrl). (**C**) Based on the LacI-miR-ON system, a firefly luciferase (Fluc) expression reporter system (LacI-miR21-ON-Fluc) activated by miR-21 was constructed and verified in HEK293 cells by co-transfecting with LacI-miR21-ON-Fluc plasmids and miRNA mimics. Then, the luciferase activity was measured 24 h later. (**D**) R21-ON or Ctrl plasmids were transfected into individual cells and the Fluc activity was measured. (**E**) The Max Fluc, wild-type LacI (LacI-WT), or mutant LacI (LacI-Mut) plasmids were transfected into HEK293 cells. Then, the leakage luciferase expression was measured. (**F-G**) Different concentrations of pLacI-T21 plasmids and a fixed dose of pLacO plasmids were transfected into HEK293 cells. (F) Western blotting was used to detect the expression of LacI protein with HA tags, and asterisks indicate a non-specific band. (G) A luciferase assay was used to detect the leakage luciferase expression of the LacI-miR21-ON-Fluc system in HEK293 cells. (**H**) Scheme for the L7Ae-miR-ON system. (**I**) L7Ae-miR21-ON-Fluc plasmids and miRNA mimics were co-transfected into HEK293 cells. Then the luciferase activity was measured 24 h later.

The LacI repression system is an inducible system established from the lac operon in *E. coli*, in which the LacI repressor suppresses gene transcription by binding to the lac operator. The LacI repression system has been widely used to construct different types of gene expression systems 17, 18. Recently, an enhanced LacI repression system was engineered by generating a tight binding LacI mutation 16. Therefore, here, a low-leakage miRNA-inducible gene expression system (LacI-miR-ON) was designed based on enhanced LacI (Figure 1A). To test the feasibility and specificity of the LacI-miR-ON system, Fluc was first chosen as the GOI and a miR21-inducible luciferase system, named LacI-miR21-ON-Fluc, was constructed (Figure 1B). LacI-miR21-ON-Fluc plasmids were transfected into HEK293 cells, which do not express endogenous miR-21, miR-9, or miR-124 19. As indicated by Fluc activity, the LacI-miR21-ON-Fluc system was activated by exogenous miR-21 mimics rather than non-target miRNA mimics, such as miR-9 or miR-124 (Figure 1C).

In addition, to test whether the LacI-miR21-ON-Fluc system could be activated by endogenous target miRNAs, a Ctrl system that did not contain miR-21 target sites compared with the LacI-miR21-ON-Fluc system was designed (Figure 1B). Ctrl or LacI-miR21-ON-Fluc (R21-ON) plasmids were transfected into several different cell types. As shown in Figure 1D, there was no significant difference in Fluc expression in HEK293 cells that did not express endogenous miR-21. In HeLa and HCT116 cells, which express high levels of endogenous miR-21 8, 20, the expression levels of Fluc were significantly higher when transfected with the R21-ON plasmids than when transfected with the Ctrl plasmids (Figure 1D). These results demonstrated that the LacI-miR21-ON-Fluc system can be activated by endogenous miR-21.

Next, the leakage expression of the LacI-miR-ON system was tested. Similar to a previous study, the enhanced lac repression (LacI-Mut) also showed substantially tighter repression than the wild-type (lacI-WT) in the LacI-miR-ON systems, although the enhanced LacI-miR-ON system still showed approximately 5% leakage of expression compared with the control Fluc (Max Fluc) (Figure 1E). It was speculated that leakage could be further inhibited by increasing LacI expression levels. However, when the LacI protein expression levels were increased (Figure 1F), sufficient LacI expression did not further inhibit the leakage of luciferase expression in the system (Figure 1G). Together, these results indicated that the LacI-miR-ON system is effective and can respond specifically to target miRNAs, but it still showed obvious leakage expression, which is similar to other reported transcriptional level regulatory systems.

### Optimization of a dual transcriptional-translational regulation system

Previous studies have reported that the coupling of transcriptional and post-transcriptional regulation could strongly suppress leakage expression in bacteria 13, 15, 21, 22. Inspired by this, it was speculated that a miRNA-responsive dual transcriptional-translational system could tighter regulate gene expression. To test this hypothesis, a miRNA-inducible translational regulatory system (L7Ae-miR-ON system) was first designed using the RNA-binding protein L7Ae (Figure 1H), which binds with high affinity to the RNA motif known as kink-turn and represses subsequent RNA translation 18, 23, 24. Using the L7Ae-miR-ON system, an miR21-inducible Fluc expression system (L7Ae-miR21-ON-Fluc) was constructed and its effectiveness and specificity were verified. As shown in Figure 1I, the luciferase signal was increased only in miR-21-transfected HEK293 cells, but not in miR-9- or miR-124-transfected cells, indicating that miR-21 could induce the activation of the L7Ae-miR21-ON-Fluc system.

**Figure 2:**
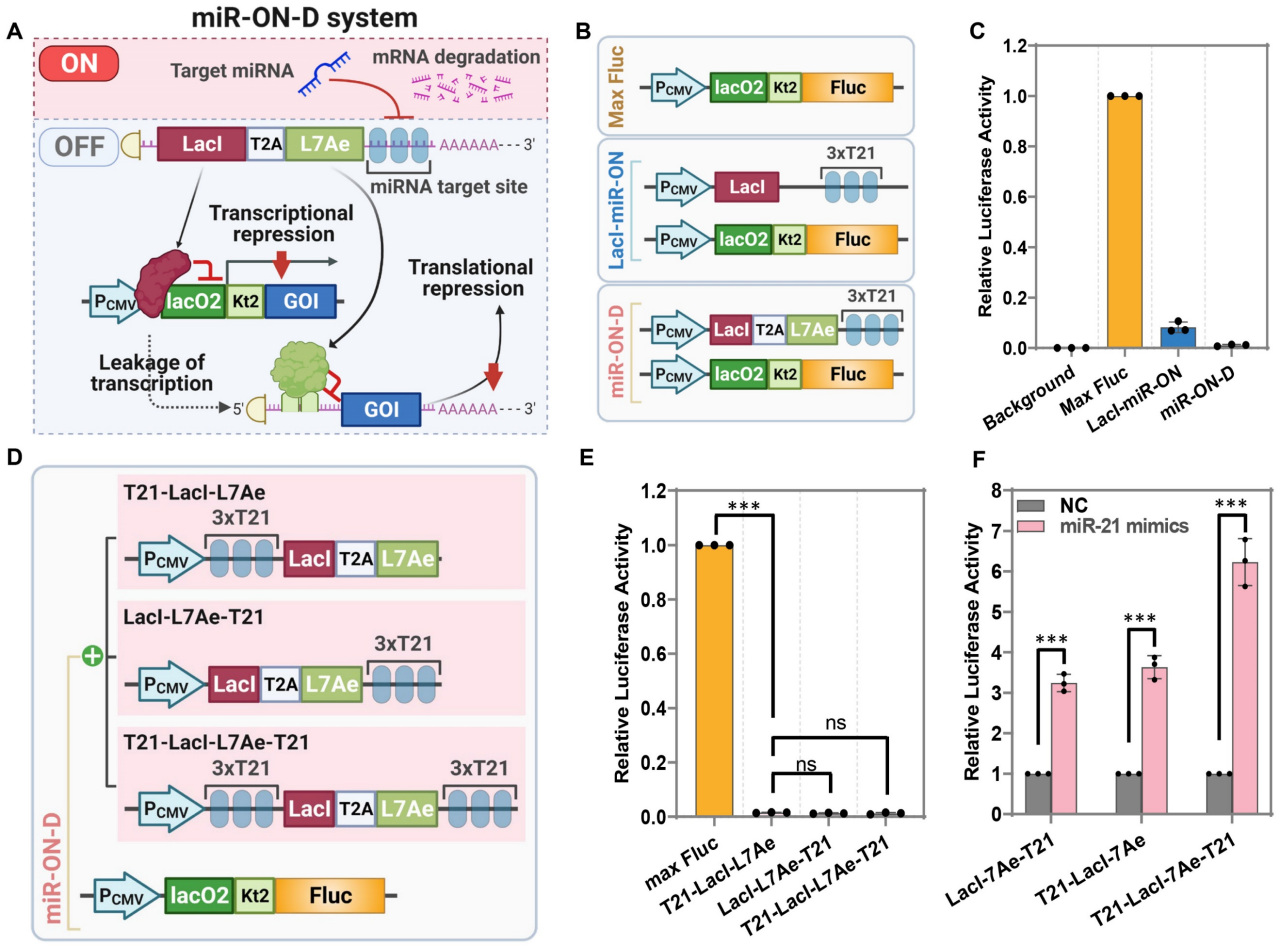
** Design and optimization of the miR-ON-D system**. (**A**) Scheme for the miR-ON-D system. The T2A sequence was used to link LacI and L7Ae. The expression of LacI inhibited the transcription of the gene of interest (GOI), while the expression of L7Ae inhibited the translation of leaked transcripts, ultimately minimizing the leakage expression. By contrast, target miRNA could inhibit the expression of LacI and L7Ae, thereby leading to the expression of the GOI. lacO2: two copies of the lac operator; kt2: two copies of the kink-turn sequence. (**B**) Scheme for the Max Fluc, LacI-miR-ON, and miR-ON-D system. (**C**) The Max Fluc, LacI-miR-ON, or miR-ON-D system plasmids were transfected in HEK293 cells to detect the leakage luciferase expression. (**D**) Scheme for three different LacI-L7Ae constructs, where miR-21 target sites were inserted into 5'-UTR (T21-LacI-L7Ae), 3'-UTR (LacI-L7Ae-T21), or both the 5′-UTR and 3′-UTR (T21-LacI-L7Ae-T21). (**E**) The three different LacI-L7Ae constructs or Max Fluc plasmids were transfected in HEK293 cells to detect the leakage luciferase expression. (**F**) Three LacI-L7Ae constructs and miRNA-21 mimics were co-transfected into HEK293 cells. Then, the luciferase activity was measured 24 h later.

Next, LacI-miR-ON was combined with the L7Ae-miR-ON system to design a miRNA-responsive dual transcriptional-translational system; the miR-ON-D system (Figure 2A). In the miR-ON-D system, LacI inhibited the transcription of a GOI, whereas L7Ae mediated translational suppression of the leaked transcripts of the GOI, ultimately minimizing leakage expression. In the presence of the target miRNA, the expression of both LacI and L7Ae is inhibited, thereby releasing the transcriptional and translational inhibition of the GOI. To test the miR-ON-D system, an miR-21 inducible Fluc expression system was constructed based on the miR-ON-D system. As expected, the miR-ON-D-based system showed extremely low leakage luciferase expression compared with LacI-miR-ON or Max Fluc (Figure 2B-C).

A previous study suggested that the insertion of miRNA target sites into both the 5′-UTR and 3′-UTR had a stronger effect than insertion into only one UTR 24. Thus, three different LacI-L7Ae constructs were designed where miR-21 target sites were inserted into the 5'-UTR (T21-LacI-L7Ae), 3'-UTR (LacI-L7Ae-T21), or both the 5′-UTR and 3′-UTR (T21-LacI-L7Ae-T21) (Figure 2D). As expected, it was found that the three forms of LacI-L7Ae constructs exhibited the same inhibitory effect on Fluc in the miR-ON-D system (Figure 2E). The miR-ON-D system containing miR-21 target sites within both the 5′-UTR and 3′-UTR (T21-LacI-L7Ae-T21) showed the highest fold-change in luciferase activity compared to standard systems containing miR-21 target sites only within the 5′-UTR (T21-L7Ae-LacI) or 3′-UTR (LacI-L7Ae-T21) after miR-21 introduction in HEK293 cells (Figure 2F). Therefore, the miR-ON-D system that contained miRNA target sites within both the 5′-UTR and 3'-UTR of LacI-L7Ae were focused on in the following studies.

### Detection of the exogenous and endogenous miRNAs in mammalian cells

**Figure 3:**
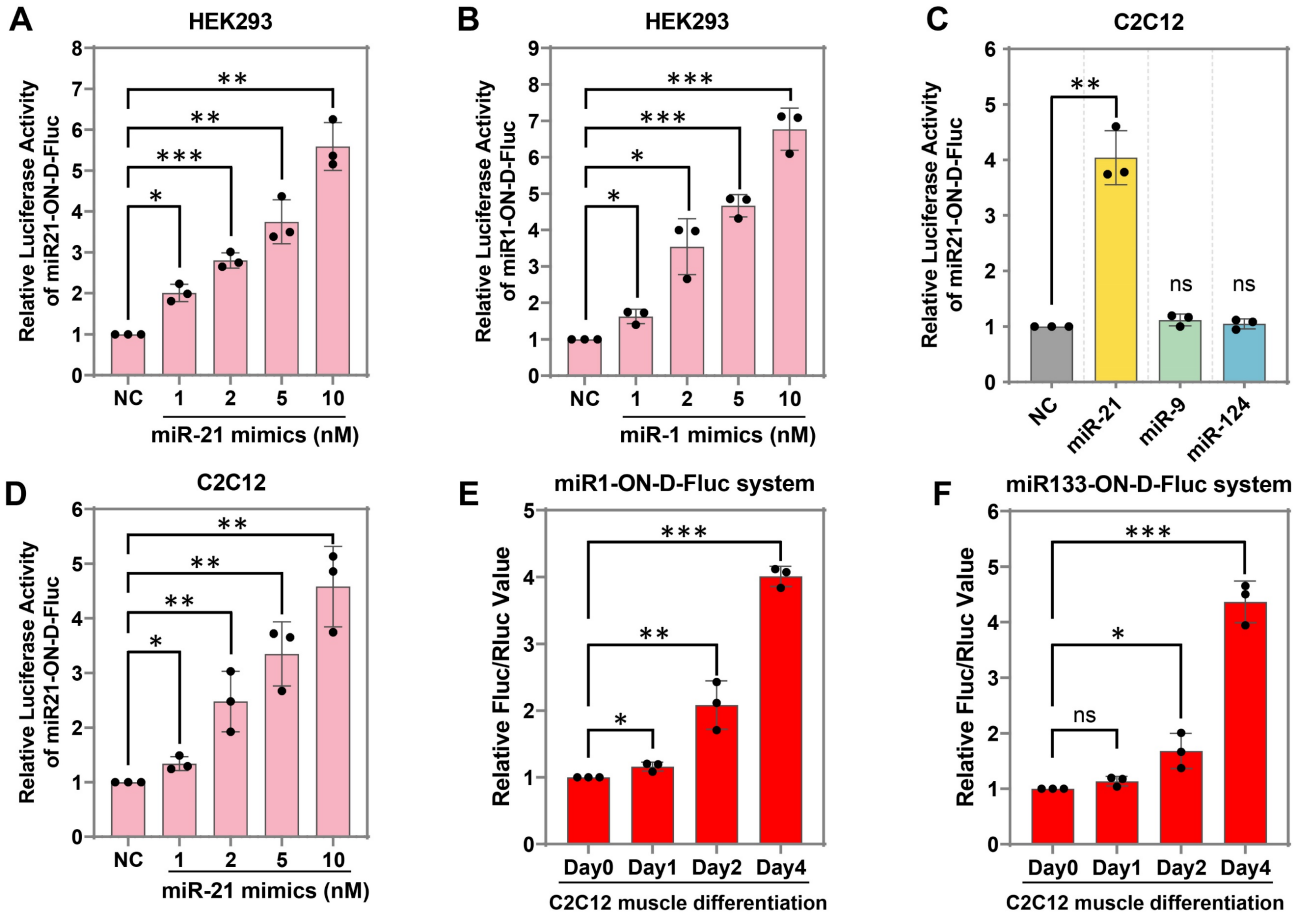
** The miR-ON-D system for exogenous and endogenous miRNA detection in mammalian cells.** (**A**) The miR21-ON-D-Fluc plasmids and different doses of miR-21 mimics were co-transfected into HEK293 cells. Then, the luciferase activity was measured 24 h later. (**B**) The miR1-ON-D-Fluc plasmids and different doses of miR-1 mimics were co-transfected into HEK293 cells. Then, the luciferase activity was measured 24 h later. (**C**) The miR21-ON-D-Fluc plasmids and different miRNA mimics (miR-21, -9, and -124) were co-transfected into C2C12 cells. Then, the luciferase activity was measured. (**D**) The miR21-ON-D-Fluc plasmids and different doses of miR-21 mimics were co-transfected into C2C12 cells. Then, the luciferase activity was measured. (**E-F**) C2C12 cells were transfected with miR1-ON-D-Fluc (E) or miR133-ON-D-Fluc (F) plasmids, then cells were shifted into a differentiation medium for a different number of days and the luciferase activity was measured.

It was expected that the miR-ON-D system could be activated by a target miRNA in a concentration-dependent manner. To test this hypothesis, we transfected miR21-ON-D-Fluc plasmids into HEK293 cells, which do not express endogenous miR-21. It was found that the expression of Fluc increased with an increasing dosage of miR-21 mimics (Figure 3A), indicating that the miR-ON-D system could be used to detect exogenous miRNAs. To test the universality of the miR-ON-D system, a miR-1-inducible Fluc expression system (miR1-ON-D-Fluc) was designed based on the miR-ON-D system. Similar to the miR21-ON-D-Fluc system, when the miR1-ON-D-Fluc plasmids and miR-1 mimics were co-transfected into HEK293 cells, the expression of Fluc increased with increasing dosage of miR-1 mimics (Figure 3B). Moreover, the miR21-ON-D-Fluc system in the C2C12 myoblast cell line was examined, which was relatively difficult to transfect. Similar to the results in HEK293 cells, miR21-ON-D-Fluc showed good specificity (Figure 3C) and was activated in a concentration-dependent manner in C2C12 cells (Figure 3D).

Next, whether the miR-ON-D system could be used to detect endogenous miRNA expression during cell differentiation was tested. For example, two muscle-specific miRNAs, miR-1 and miR-133, were chosen, the expressions of which were apparent in differentiated myotubes but not in myoblasts 25. A miR-1- or miR-133-inducible Fluc expression system was constructed based on the miR-ON-D system, named the miR1-ON-D-Fluc or miR133-ON-D-Fluc system, respectively. Notably, when miR1-ON-D-Fluc (Figure 3E) or miR133-ON-D-Fluc (Figure 3F) plasmids were transfected into C2C12 cells, Fluc activity gradually increased over time during C2C12 cell differentiation, indicating that upregulated miR-1 or miR-133 activated Fluc expression. Together, these results demonstrated that the miR-ON-D system can be used as a reporter to detect both exogenous and endogenous miRNA expression.

### The miR-ON-D system inhibited the proliferation of specific cell types

**Figure 4:**
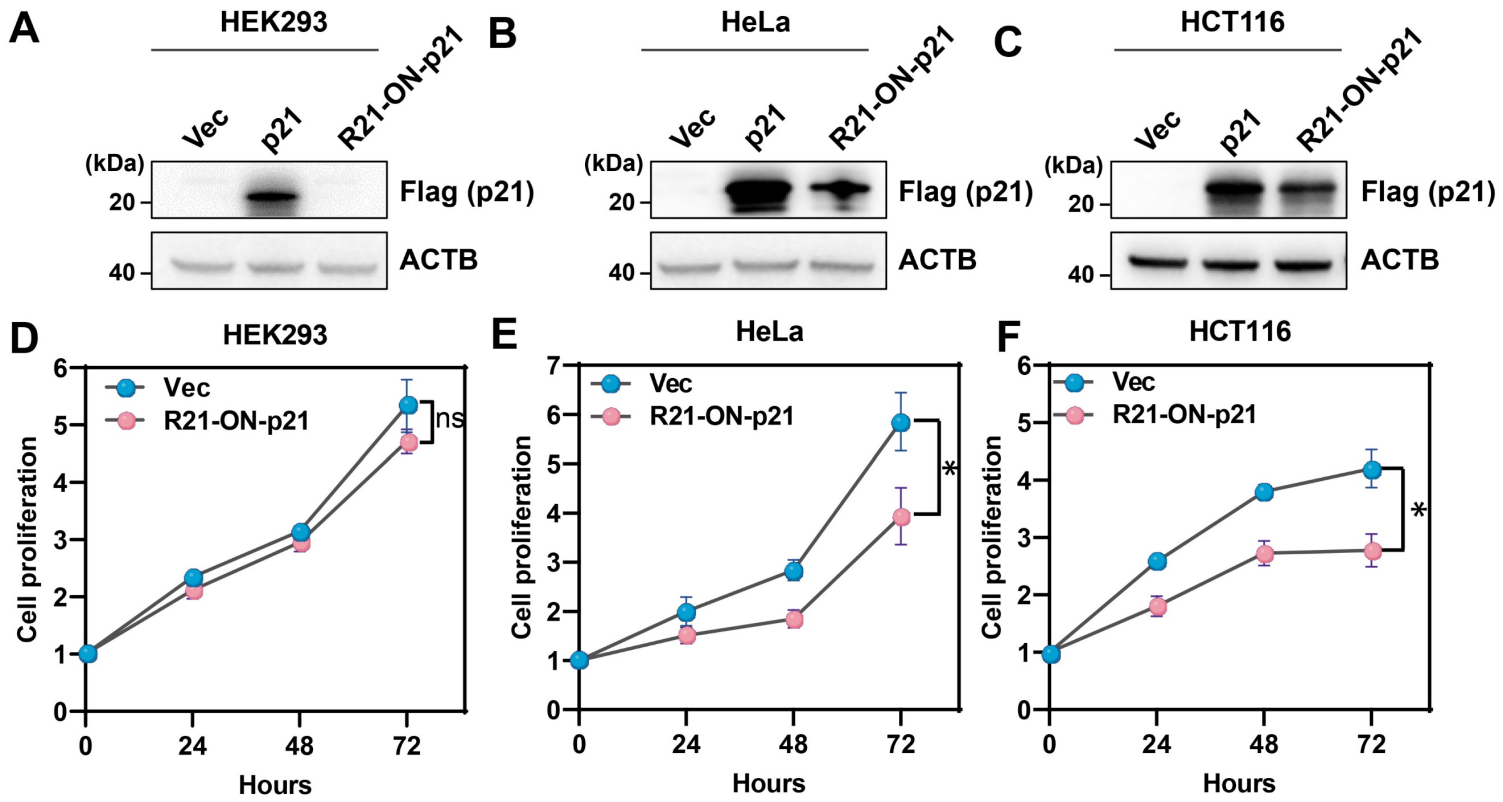
** The miR-ON-D system could be used to efficiently inhibit the proliferation of target cells.** (**A-C**) The empty vector (Vec), positive control plasmid expressing p21 protein (p21), or R21-ON-p21 plasmids with Flag tags were transfected in (A) HEK293, (B) HeLa, or (C) HCT116 cells. The exogenous p21 protein levels were detected using Flag antibody 48 h later. β-Actin (ACTB) served as a loading control. (**D-F**) Proliferation of the transfected (**D**) HEK293, (**E**) HeLa, or (**F**) HCT116 cells was measured using a CCK-8 assay at different time intervals. The results at each time point are shown as the mean ± SD. Each experiment was independently performed three times.

Next, whether the miR-ON-D system could be triggered by cell type-specific miRNAs to regulate the expression of biologically relevant proteins was explored. In this case, p21 (also known as CDKN1, CAP20, or CIP1) was chosen, which suppresses cell proliferation by inhibiting the cell cycle through the cyclin kinase pathway 26, 27. A miR-21-inducible p21 expression system (R21-ON-p21) was constructed by replacing the luciferase gene with p21 in the miR21-ON-D-Fluc system. The R21-ON-p21 system was tested in HEK293 cells, which did not express endogenous miR21, and in HeLa and HCT116 cells, which expressed high levels of miR-21. As shown in Figure 4A-C, exogenous p21 protein with FLAG tags (positive control) could be expressed in these three cell types. However, when transfecting the R21-ON-p21 plasmids into these three different cell types, exogenous p21 was only detected in HeLa and HCT116 cells, indicating that endogenous miR21 activated p21 expression.

The CCK-8 assay was performed to determine cell proliferation at various time points. As shown in Figure 4D, there was no significant difference in cell proliferation between the empty vector- and R21-ON-p21 plasmid-transfected HEK293 cells. In contrast, when the R21-ON-p21 plasmid was transfected into HeLa (Figure 4E) or HCT116 cells (Figure 4F), cell proliferation was significantly inhibited compared with that of the empty vector. Taken together, these results demonstrated that the miR-ON-D system could be employed to efficiently inhibit the proliferation of specific cell types by activating p21 gene expression.

### The miR-ON-D system induced cell type-specific apoptotic death

To further extend the applicability of the miR-ON-D system, a miR-21-inducible Bax expression system was designed based on the miR-ON-D system named the R21-ON-Bax system. Bax is a member of the Bcl-2 protein family and is associated with apoptotic cell death in both cell cultures and intact animals. Overexpression of Bax in cells can destroy the mitochondrial transmembrane potential and lead to the release of cytochrome c, which leads to apoptosis 28. Previous studies have shown that low levels of leaky expression are required for inducible expression of toxic proteins 15. In the present study's miR-ON-D system, leakage expression was strongly suppressed. Therefore, it is suspected that the R21-ON-Bax system functions as follows. In non-target cells lacking miR-21, Bax expression is almost completely inhibited, thereby producing no toxicity in cells. Bax is expressed in target cells expressing miR-21 at high levels, thereby causing cell death.

**Figure 5:**
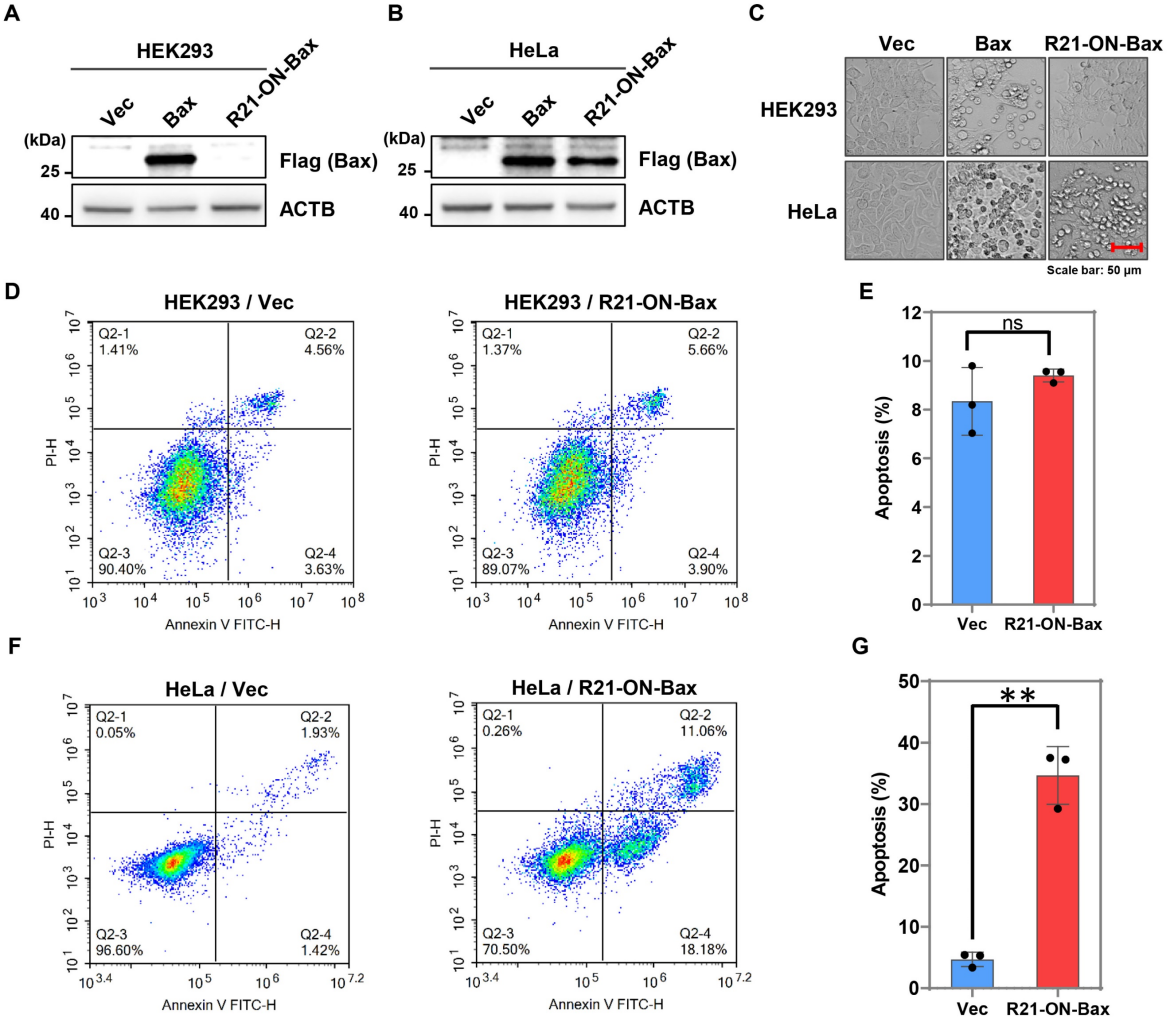
** Cell type-specific apoptotic cell death induced by the miR-ON-D system.** (**A, B**) The empty vector (Vec), control plasmid expressing Bax protein (Bax), or R21-ON-Bax plasmids with Flag tags were transfected in (**A**) HEK293 or (**B**) HeLa cells. The expression of the Bax protein was detected using the Flag antibody 48 h later. β-Actin (ACTB) served as a loading control. (**C**) The cell morphology under different transfection conditions was as above. (**D-E**) Flow cytometric analyses and quantification of cell apoptosis in HEK293 cells transfected with vector or R21-ON-Bax plasmids. (**F-G**) Flow cytometric analyses and quantification of cell apoptosis of HeLa cells transfected with vector or R21-ON-Bax plasmids.

To test this hypothesis, the R21-ON-Bax system was tested in HEK293 cells (non-target cells), which do not express endogenous miR21, and HeLa cells (target cells), which express high levels of miR-21. As shown in Figure 5A-B, exogenous Bax protein with FLAG tags can be expressed in both HEK293 and HeLa cells. In contrast, when the R21-ON-Bax plasmids were transfected into these two cell types, the expression of the Bax protein was detected only in HeLa cells, indicating that endogenous miR21 induced the activation of Bax. Importantly, the results showed that the R21-ON-Bax plasmid led to apparent cell death in HeLa cells, but not in HEK293 cells (Figure 5C). In addition, to further determine whether the R21-ON-Bax system could selectively kill target cells, flow cytometry was performed to detect cell apoptosis under different conditions. As shown in Figure 5D-E, in HEK293 cells, the expression of the R21-ON-Bax plasmid did not induce more apoptosis compared with the empty vector. In contrast, the introduction of the R21-ON-Bax plasmids in HeLa cells resulted in a higher proportion of cell apoptosis than that in the vector control group (Figure 5F-G). Together, these results demonstrated that the R21-ON-Bax system could specifically and efficiently kill cells with high miR21 expression, revealing the superiority and cell type targeting of the miR-ON-D system.

## Conclusion

In this study, a tight miRNA-inducible gene expression switch system (miR-ON-D) was successfully designed by adopting a dual transcriptional-translational control strategy. In the miR-ON-D system, the GOI was tightly controlled and could be activated by a cell type-specific miRNA. Using the miR-ON-D switch system, the expression of both exogenous and endogenous miRNAs was successfully detected in mammalian cells. Moreover, the miR-ON-D system could effectively inhibit proliferation or induce apoptotic death of specific cell types by activating p21 or Bax expression. This study demonstrated the capability of miR-ON-D in miRNA sensing and the cell type-specific control of biologically relevant gene expression, which may provide a promising strategy for RNA imaging and synthetic biology applications.

## Supplementary Material

Supplementary tables.
