# Supplementary material for: A dual-regulation inducible switch system for microRNA detection and cell type-specific gene activation
Source: Theranostics. 2023 Apr 23;13(8):2552–61. doi: 10.7150/thno.84111 (PMC10196832; doi:10.7150/thno.84111)
Supplement: Supplementary file 1 — Supplementary tables. [file thnov13p2552s1.pdf]

Supporting information for

A dual-regulation inducible switch system for microRNA detection and cell type-specific gene activation

Wen-Jie Shu[1], Kyungwoo Lee[2], Zhe Ma[1], Xiaojie Tian[1], Jong Seung Kim[2]*
and Fu Wang[1, 3, 4]*

[1] School of Basic Medical Sciences, Xi'an Jiaotong University, Xi'an 710061, China.

[2] Department of Chemistry, Korea University, Seoul 02841, Korea.

[3] Engineering Research Center of Molecular and Neuro Imaging, Ministry of Education, School of Life Science and Technology, Xidian University, Xi'an 710071, China.

[4] Xianyang Key Laboratory of Molecular Imaging and Drug Synthesis, School of Pharmacy, Shaanxi Institute of International Trade & Commerce, Xianyang 712046, Shaanxi, China

*To whom correspondence should be addressed:

Fu Wang, E-mail: wangfu@xjtu.edu.cn

Jong Seung Kim, E-mail: jongskim@korea.ac.kr

Supplementary Table 1. DNA sequence information of plasmids used in this study

LacI-HA-3xT21
ATGGGCTACGTAACGTTATACGATGTCGCAGAGTATGCCGGTGTCTCTTATCAGACCGT<br>TTCCCGCGTGGTGAACCAGGCCAGCCACGTTTCTGCGAAAACGCGGGAAAAAGTGGA<br>AGCGGCGATGGCGGAGCTGAATTACATTCCCAACCGCGTGGCACAACAACTGGCGGG<br>CAAACAGTCGTTGCTGATTGGCGTTGCCACCTCCAGTCTGGCCCTGCACGCGCCGTC<br>GCAAATTGTCGCGGCGATTAAATCTCGCGCCGATCAACTGGGTGCCAGCGTGGTGGT<br>GTCGATGGTAGAACGAAGCGGCGTCGAAGCCTGTAAAGCGGCGGTGCACAATCTTCT<br>CGCGCAACGCGTCAGTGGGCTGATCATTAACTATCCGCTGGATGACCAGGATGCCATT<br>GCTGTGGAAGCTGCCTGCACTAATGTTCCGGCGTTATTTCTTGATGTCTCTGACCAGA<br>CACCCATCAACAGTATTATTTTCTCCCATGAAGACGGTACGCGACTGGGCGTGGAGCA<br>TCTGGTCGCATTGGGTCACCAGCAAATCGCGCTGTTAGCGGGCCCATTAAGTTCTGTC<br>TCGGCGCGTCTGCGTCTGGCTGGCTGGCATAAATATCTCACTCGCAATCAAATTCAGC<br>CGATAGCGGAACGGGAAGGCGACTGGAGTGCCATGTCCGGTTTTCAACAAACCATGC<br>AAATGCTGAATGAGGGCATCGTTCCCACTGCGATGCTGGTTGCCAACGATCAGATGGC<br>GCTGGGCGCAATGCGCGCCATTACCGAGTCCGGGCTGCGCGTTGGTGCGGATATCTC<br>GGTAGTGGGATACGACGATACCGAAGACAGCTCATGTTATATCCCGCCGTTAACCACCA<br>TCAAACAGGATTTTCGCCTGCTGGGGCAAACCAGCGTGGACCGCTTGCTGCAACTCT<br>CTCAGGGCCAGGCGGTGAAGGGCAATCAGCTGTTGCCCGTCTCACTGGTGAAAAGAA<br>AAACCACCCTGGCGCCCAATACGCAAACCGCCTCTCCCCGCGCGTTGGCCGATTCAT<br>TAATGCAGCTGGCACGACAGGTTTCCCGACTGGAAAGCGGGCAGGAATTCCCTAAGA<br>AGAAGAGGAAGGTTCTCGAGTACCCATACGATGTTCCAGATTACGCTTGAGAATTCACG<br>CGTCAACATCAGTCTGATAAGCTATCAACATCAGTCTGATAAGCTATCAACATCAGTCTG<br>ATAAGCTA
**LacO2-Fluc**
AATTGTGAGCGCTCACAATTCCCACAGTCGACCCTAGGTTGTGGAATTGTGAGCGCTC<br>ACAATTGAATTCACGCGTGGTACCTCTAGAGCCACC**ATGGCCGATGCTAAGAACATTA**<br>**AGAAGGGCCCTGCTCCCTTCTACCCTCTGGAGGATGGCACCGCTGGCGAGCAGCTG**<br>**CACAAGGCCATGAAGAGGTATGCCCTGGTGCCTGGCACCATTGCCTTCACCGATGC**<br>**CCACATTGAGGTGGACATCACCTATGCCGAGTACTTCGAGATGTCTGTGCGCCTGGC**<br>**CGAGGCCATGAAGAGGTACGGCCTGAACACCAACCACCGCATCGTGGTGTGCTCTG**<br>**AGAACTCTCTGCAGTTCTTCATGCCAGTGCTGGGCGCCCTGTTCATCGGAGTGGCCG**<br>**TGGCCCCTGCTAACGACATTTACAACGAGCGCGAGCTGCTGAACAGCATGGGCATTT**<br>**CTCAGCCTACCGTGGTGTTCGTGTCTAAGAAGGGCCTGCAGAAGATCCTGAACGTG**<br>**CAGAAGAAGCTGCCTATCATCCAGAAGATCATCATCATGGACTCTAAGACCGACTAC**<br>**CAGGGCTTCCAGAGCATGTACACATTCGTGACATCTCATCTGCCTCCTGGCTTCAAC**<br>**GAGTACGACTTCGTGCCAGAGTCTTTCGACAGGGACAAAACCATTGCCCTGATCATG**<br>**AACAGCTCTGGGTCTACCGGCCTGCCTAAGGGCGTGGCCCTGCCTCATCGCACCGC**<br>**CTGTGTGCGCTTCTCTCACGCCCGCGACCCTATTTTCGGCAACCAGATCATCCCCGA**<br>**CACCGCTATTCTGAGCGTGGTGCCATTCCACCACGGCTTCGGCATGTTCACCACCCT**<br>**GGGCTACCTGATTTGCGGCTTTCGGGTGGTGCTGATGTACCGCTTCGAGGAGGAGCT**<br>**GTTCCTGCGCAGCCTGCAAGACTACAAAATTCAGTCTGCCCTGCTGGTGCCAACCCT**

**GTTCAGCTTCTTCGCTAAGAGCACCCTGATCGACAAGTACGACCTGTCTAACCTGCA<br>CGAGATTGCCTCTGGCGGCGCCCCACTGTCTAAGGAGGTGGGCGAAGCCGTGGCC<br>AAGCGCTTTCATCTGCCAGGCATCCGCCAGGGCTACGGCCTGACCGAGACAACCAG<br>CGCCATTCTGATTACCCCAGAGGGCGACGACAAGCCTGGCGCCGTGGGCAAGGTG<br>GTGCCATTCTTCGAGGCCAAGGTGGTGGACCTGGACACCGGCAAGACCCTGGGAGT<br>GAACCAGCGCGGCGAGCTGTGTGTGCGCGGCCCTATGATTATGTCCGGCTACGTGA<br>ATAACCCTGAGGCCACAAACGCCCTGATCGACAAGGACGGCTGGCTGCACTCTGGC<br>GACATTGCCTACTGGGACGAGGACGAGCACTTCTTCATCGTGGACCGCCTGAAGTCT<br>CTGATCAAGTACAAGGGCTACCAGGTGGCCCCAGCCGAGCTGGAGTCTATCCTGCT<br>GCAGCACCCTAACATTTTCGACGCCGGAGTGGCCGGCCTGCCCGACGACGATGCCG<br>GCGAGCTGCCTGCCGCCGTCGTCGTGCTGGAACACGGCAAGACCATGACCGAGAA<br>GGAGATCGTGGACTATGTGGCCAGCCAGGTGACAACCGCCAAGAAGCTGCGCGGC<br>GGAGTGGTGTTCGTGGACGAGGTGCCCAAGGGCCTGACCGGCAAGCTGGACGCCC<br>GCAAGATCCGCGAGATCCTGATCAAGGCTAAGAAAGGCGGCAAGATCGCCGTGTCT<br>CGAGTCGACGACTACAAAGACCATGACGGTGATTATAAAGATCATGACATCGACTAC<br>AAGGATGACGATGACAAGTAA**
**L7Ae-3xT21**
**ATGTACGTGAGATTTGAGGTTCCTGAGGACATGCAGAACGAAGCTCTGAGTCTGCTG<br>GAGAAGGTTAGGGAGAGCGGTAAGGTAAAGAAAGGTACCAACGAGACGACAAAGG<br>CTGTGGAGAGGGGACTGGCAAAGCTCGTTTACATCGCAGAGGATGTTGACCCGCCT<br>GAGATCGTTGCTCATCTGCCCCTCCTCTGCGAGGAGAAGAATGTGCCGTACATTTAC<br>GTTAAAAGCAAGAACGACCTTGGAAGGGCTGTGGGCATTGAGGTGCCATGCGCTTC<br>GGCAGCGATAATCAACGAGGGAGAGCTGAGAAAGGAGCTTGGAAGCCTTGTGGAG<br>AAGATTAAAGGCCTTCAGAAGAGATCTCATATGCATCTCGAGTACCCATACGATGTTC<br>CAGATTACGCTTGA**GAATTCACGCGTCAACATCAGTCTGATAAGCTATCAACATCAGTC<br>TGATAAGCTATCAACATCAGTCTGATAAGCTA
LacO2-Kt2-**Fluc**
AATTGTGAGCGCTCACAATTCCCACAGTCGACCCTAGGTTGTGGAATTGTGAG<br>CGCTCACAATTGGGCGTGATCCGAAAGGTGACCCGAATTGGGCGTGATCCGA<br>AAGGTGACCCTAGTCCAGTGTGGTGGAATTCTCTAGAGCCACC**ATGGCCGAT<br>GCTAAGAACATTAAGAAGGGCCCTGCTCCCTTCTACCCTCTGGAGGATGGCA<br>CCGCTGGCGAGCAGCTGCACAAGGCCATGAAGAGGTATGCCCTGGTGCCTG<br>GCACCATTGCCTTCACCGATGCCCACATTGAGGTGGACATCACCTATGCCGA<br>GTACTTCGAGATGTCTGTGCGCCTGGCCGAGGCCATGAAGAGGTACGGCCT<br>GAACACCAACCACCGCATCGTGGTGTGCTCTGAGAACTCTCTGCAGTTCTTC<br>ATGCCAGTGCTGGGCGCCCTGTTCATCGGAGTGGCCGTGGCCCCTGCTAAC<br>GACATTTACAACGAGCGCGAGCTGCTGAACAGCATGGGCATTTCTCAGCCTA<br>CCGTGGTGTTCGTGTCTAAGAAGGGCCTGCAGAAGATCCTGAACGTGCAGA<br>AGAAGCTGCCTATCATCCAGAAGATCATCATCATGGACTCTAAGACCGACTA<br>CCAGGGCTTCCAGAGCATGTACACATTCGTGACATCTCATCTGCCTCCTGGC<br>TTCAACGAGTACGACTTCGTGCCAGAGTCTTTCGACAGGGACAAAACCATT<br>GCCCTGATCATGAACAGCTCTGGGTCTACCGGCCTGCCTAAGGGCGTGGCC**

**CTGCCTCATCGCACCGCCTGTGTGCGCTTCTCTCACGCCCGCGACCCTATTT
TCGGCAACCAGATCATCCCCGACACCGCTATTCTGAGCGTGGTGCCATTCCA
CCACGGCTTCGGCATGTTCACCACCCTGGGCTACCTGATTTGCGGCTTTCGG
GTGGTGCTGATGTACCGCTTCGAGGAGGAGCTGTTCCTGCGCAGCCTGCAA
GACTACAAAATTCAGTCTGCCCTGCTGGTGCCAACCCTGTTCAGCTTCTTCG
CTAAGAGCACCCTGATCGACAAGTACGACCTGTCTAACCTGCACGAGATTGC
CTCTGGCGGCGCCCCACTGTCTAAGGAGGTGGGCGAAGCCGTGGCCAAGC
GCTTTCATCTGCCAGGCATCCGCCAGGGCTACGGCCTGACCGAGACAACCA
GCGCCATTCTGATTACCCCAGAGGGCGACGACAAGCCTGGCGCCGTGGGC
AAGGTGGTGCCATTCTTCGAGGCCAAGGTGGTGGACCTGGACACCGGCAA
GACCCTGGGAGTGAACCAGCGCGGCGAGCTGTGTGTGCGCGGCCCTATGAT
TATGTCCGGCTACGTGAATAACCCTGAGGCCACAAACGCCCTGATCGACAA
GGACGGCTGGCTGCACTCTGGCGACATTGCCTACTGGGACGAGGACGAGC
ACTTCTTCATCGTGGACCGCCTGAAGTCTCTGATCAAGTACAAGGGCTACCA
GGTGGCCCCAGCCGAGCTGGAGTCTATCCTGCTGCAGCACCCTAACATTTTC
GACGCCGGAGTGGCCGGCCTGCCCGACGACGATGCCGGCGAGCTGCCTGC
CGCCGTCGTCGTGCTGGAACACGGCAAGACCATGACCGAGAAGGAGATCG
TGGACTATGTGGCCAGCCAGGTGACAACCGCCAAGAAGCTGCGCGGCGGA
GTGGTGTTCGTGGACGAGGTGCCCAAGGGCCTGACCGGCAAGCTGGACGC
CCGCAAGATCCGCGAGATCCTGATCAAGGCTAAGAAAGGCGGCAAGATCGC
CGTGCTCGAGCTCGACGACTACAAAGACCATGACGGTGATTATAAAGATCAT
GACATCGACTACAAGGATGACGATGACAAGTAA**

3xT21-LacI-T2A-L7Ae-3xT21

TCAACATCAGTCTGATAAGCTATCAACATCAGTCTGATAAGCTATCAACATCAGT
CTGATAAGCTATCTAGAGCCACCATGGCTACGTAACGTTATACGATGTCGCAG
AGTATGCCGGTGTCTCTTATCAGACCGTTTCCCGCGTGGTGAACCAGGCCAG
CCACGTTTCTGCGAAAACGCGGGAAAAAGTGGAAGCGGCGATGGCGGAGCT
GAATTACATTCCCAACCGCGTGGCACAACAACTGGCGGGCAAACAGTCGTTG
CTGATTGGCGTTGCCACCTCCAGTCTGGCCCTGCACGCGCCGTCGCAAATTG
TCGCGGCGATTAAATCTCGCGCCGATCAACTGGGTGCCAGCGTGGTGGTGTC
GATGGTAGAACGAAGCGGCGTCGAAGCCTGTAAAGCGGCGGTGCACAATCTT
CTCGCGCAACGCGTCAGTGGGCTGATCATTAACTATCCGCTGGATGACCAGG
ATGCCATTGCTGTGGAAGCTGCCTGCACTAATGTTCCGGCGTTATTTCTTGATG
TCTCTGACCAGACACCCATCAACAGTATTATTTTCTCCCATGAAGACGGTACGC
GACTGGGCGTGGAGCATCTGGTCGCATTGGGTCACCAGCAAATCGCGCTGTT
AGCGGGCCCATTAAGTTCTGTCTCGGCGCGTCTGCGTCTGGCTGGCTGGCAT
AAATATCTCACTCGCAATCAAATTCAGCCGATAGCGGAACGGGAAGGCGACTG
GAGTGCCATGTCCGGTTTTCAACAAACCATGCAAATGCTGAATGAGGGCATCG
TTCCCACTGCGATGCTGGTTGCCAACGATCAGATGGCGCTGGGCGCAATGCG
CGCCATTACCGAGTCCGGGCTGCGCGTTGGTGCGGATATCTCGGTAGTGGGA
TACGACGATACCGAAGACAGCTCATGTTATATCCCGCCGTTAACCACCATCAAA
CAGGATTTTCGCCTGCTGGGGCAAACCAGCGTGGACCGCTTGCTGCAACTCT
CTCAGGGCCAGGCGGTGAAGGGCAATCAGCTGTTGCCCGTCTCACTGGTGA

AAAGAAAAACCACCCTGGCGCCCAATACGCAAACCGCCTCTCCCCGCGCGTT<br>GGCCGATTCATTAATGCAGCTGGCACGACAGGTTTCCCGACTGGAAAGCGGG<br>CAGGAATTCCCTAAGAAGAAGAGGAAGGTTGGGAGCGGCGCCACAAACTTTT<br>CCCTCCTGAAGCAGGCTGGAGATGTGGAGGAGAATCCCGGACCTAGCGGAAT<br>GTACGTGAGATTTGAGGTTCCTGAGGACATGCAGAACGAAGCTCTGAGTCTG<br>CTGGAGAAGGTTAGGGAGAGCGGTAAGGTAAAGAAAGGTACCAACGAGACGA<br>CAAAGGCTGTGGAGAGGGGACTGGCAAAGCTCGTTTACATCGCAGAGGATGT<br>TGACCCGCCTGAGATCGTTGCTCATCTGCCCCTCCTCTGCGAGGAGAAGAAT<br>GTGCCGTACATTTACGTTAAAAGCAAGAACGACCTTGGAAGGGCTGTGGGCAT<br>TGAGGTGCCATGCGCTTCGGCAGCGATAATCAACGAGGGAGAGCTGAGAAAG<br>GAGCTTGGAAGCCTTGTGGAGAAGATTAAAGGCCTTCAGAAGAGATCTCATAT<br>GCATCTCGAGTACCCATACGATGTTCCAGATTACGCTTGAGAATTCACGCGTCA<br>ACATCAGTCTGATAAGCTATCAACATCAGTCTGATAAGCTATCAACATCAGTCTG<br>ATAAGCTA
LacI-T2A-L7Ae-3xT21
ATGGGCTACGTAACGTTATACGATGTCGCAGAGTATGCCGGTGTCTCTTATCAG<br>ACCGTTTCCCGCGTGGTGAACCAGGCCAGCCACGTTTCTGCGAAAACGCGG<br>GAAAAAGTGGAAGCGGCGATGGCGGAGCTGAATTACATTCCCAACCGCGTGG<br>CACAACAACTGGCGGGCAAACAGTCGTTGCTGATTGGCGTTGCCACCTCCAG<br>TCTGGCCCTGCACGCGCCGTCGCAAATTGTCGCGGCGATTAAATCTCGCGCC<br>GATCAACTGGGTGCCAGCGTGGTGGTGTCGATGGTAGAACGAAGCGGCGTC<br>GAAGCCTGTAAAGCGGCGGTGCACAATCTTCTCGCGCAACGCGTCAGTGGG<br>CTGATCATTAACTATCCGCTGGATGACCAGGATGCCATTGCTGTGGAAGCTGC<br>CTGCACTAATGTTCCGGCGTTATTTCTTGATGTCTCTGACCAGACACCCATCAA<br>CAGTATTATTTTCTCCCATGAAGACGGTACGCGACTGGGCGTGGAGCATCTGG<br>TCGCATTGGGTCACCAGCAAATCGCGCTGTTAGCGGGCCCATTAAGTTCTGTC<br>TCGGCGCGTCTGCGTCTGGCTGGCTGGCATAAATATCTCACTCGCAATCAAAT<br>TCAGCCGATAGCGGAACGGGAAGGCGACTGGAGTGCCATGTCCGGTTTTCAA<br>CAAACCATGCAAATGCTGAATGAGGGCATCGTTCCCACTGCGATGCTGGTTGC<br>CAACGATCAGATGGCGCTGGGCGCAATGCGCGCCATTACCGAGTCCGGGCT<br>GCGCGTTGGTGCGGATATCTCGGTAGTGGGATACGACGATACCGAAGACAGC<br>TCATGTTATATCCCGCCGTTAACCACCATCAAACAGGATTTTCGCCTGCTGGGG<br>CAAACCAGCGTGGACCGCTTGCTGCAACTCTCTCAGGGCCAGGCGGTGAAG<br>GGCAATCAGCTGTTGCCCGTCTCACTGGTGAAAAGAAAAACCACCCTGGCGC<br>CCAATACGCAAACCGCCTCTCCCCGCGCGTTGGCCGATTCATTAATGCAGCTG<br>GCACGACAGGTTTCCCGACTGGAAAGCGGGCAGGAATTCCCTAAGAAGAAGA<br>GGAAGGTTGGGAGCGGCGCCACAAACTTTTCCCTCCTGAAGCAGGCTGGAG<br>ATGTGGAGGAGAATCCCGGACCTAGCGGAATGTACGTGAGATTTGAGGTTCCT<br>GAGGACATGCAGAACGAAGCTCTGAGTCTGCTGGAGAAGGTTAGGGAGAGC<br>GGTAAGGTAAAGAAAGGTACCAACGAGACGACAAAGGCTGTGGAGAGGGGA<br>CTGGCAAAGCTCGTTTACATCGCAGAGGATGTTGACCCGCCTGAGATCGTTG<br>CTCATCTGCCCCTCCTCTGCGAGGAGAAGAATGTGCCGTACATTTACGTTAAA<br>AGCAAGAACGACCTTGGAAGGGCTGTGGGCATTGAGGTGCCATGCGCTTCG

GCAGCGATAATCAACGAGGGAGAGCTGAGAAAGGAGCTTGGAAGCCTTGTGG<br>AGAAGATTAAAGGCCTTCAGAAGAGATCTCATATGCATCTCGAGTACCCATACG<br>ATGTTCCAGATTACGCTTGAGAATTCACGCGTCAACATCAGTCTGATAAGCTAT<br>CAACATCAGTCTGATAAGCTATCAACATCAGTCTGATAAGCTA
3xT21-LacI-T2A-L7Ae
TCAACATCAGTCTGATAAGCTATCAACATCAGTCTGATAAGCTATCAACATCAGT<br>CTGATAAGCTATCTAGAGCCACCATGGCTACGTAACGTTATACGATGTCGCAG<br>AGTATGCCGGTGTCTCTTATCAGACCGTTTCCCGCGTGGTGAACCAGGCCAG<br>CCACGTTTCTGCGAAAACGCGGGAAAAAGTGGAAGCGGCGATGGCGGAGCT<br>GAATTACATTCCCAACCGCGTGGCACAACAACTGGCGGGCAAACAGTCGTTG<br>CTGATTGGCGTTGCCACCTCCAGTCTGGCCCTGCACGCGCCGTCGCAAATTG<br>TCGCGGCGATTAAATCTCGCGCCGATCAACTGGGTGCCAGCGTGGTGGTGTC<br>GATGGTAGAACGAAGCGGCGTCGAAGCCTGTAAAGCGGCGGTGCACAATCTT<br>CTCGCGCAACGCGTCAGTGGGCTGATCATTAACTATCCGCTGGATGACCAGG<br>ATGCCATTGCTGTGGAAGCTGCCTGCACTAATGTTCCGGCGTTATTTCTTGATG<br>TCTCTGACCAGACACCCATCAACAGTATTATTTTCTCCCATGAAGACGGTACGC<br>GACTGGGCGTGGAGCATCTGGTCGCATTGGGTCACCAGCAAATCGCGCTGTT<br>AGCGGGCCCATTAAGTTCTGTCTCGGCGCGTCTGCGTCTGGCTGGCTGGCAT<br>AAATATCTCACTCGCAATCAAATTCAGCCGATAGCGGAACGGGAAGGCGACTG<br>GAGTGCCATGTCCGGTTTTCAACAAACCATGCAAATGCTGAATGAGGGCATCG<br>TTCCCACTGCGATGCTGGTTGCCAACGATCAGATGGCGCTGGGCGCAATGCG<br>CGCCATTACCGAGTCCGGGCTGCGCGTTGGTGCGGATATCTCGGTAGTGGGA<br>TACGACGATACCGAAGACAGCTCATGTTATATCCCGCCGTTAACCACCATCAAA<br>CAGGATTTTCGCCTGCTGGGGCAAACCAGCGTGGACCGCTTGCTGCAACTCT<br>CTCAGGGCCAGGCGGTGAAGGGCAATCAGCTGTTGCCCGTCTCACTGGTGA<br>AAAGAAAAACCACCCTGGCGCCCAATACGCAAACCGCCTCTCCCCGCGCGTT<br>GGCCGATTCATTAATGCAGCTGGCACGACAGGTTTCCCGACTGGAAAGCGGG<br>CAGGAATTCCCTAAGAAGAAGAGGAAGGTTGGGAGCGGCGCCACAAACTTTT<br>CCCTCCTGAAGCAGGCTGGAGATGTGGAGGAGAATCCCGGACCTAGCGGAAT<br>GTACGTGAGATTTGAGGTTCCTGAGGACATGCAGAACGAAGCTCTGAGTCTG<br>CTGGAGAAGGTTAGGGAGAGCGGTAAGGTAAAGAAAGGTACCAACGAGACGA<br>CAAAGGCTGTGGAGAGGGGACTGGCAAAGCTCGTTTACATCGCAGAGGATGT<br>TGACCCGCCTGAGATCGTTGCTCATCTGCCCCTCCTCTGCGAGGAGAAGAAT<br>GTGCCGTACATTTACGTTAAAAGCAAGAACGACCTTGGAAGGGCTGTGGGCAT<br>TGAGGTGCCATGCGCTTCGGCAGCGATAATCAACGAGGGAGAGCTGAGAAAG<br>GAGCTTGGAAGCCTTGTGGAGAAGATTAAAGGCCTTCAGAAGAGATCTCATAT<br>GCATCTCGAGTACCCATACGATGTTCCAGATTACGCTTGA
3xT1-LacI-T2A-L7Ae-3xT1
ATACATACTTCTTTACATTCCAATACATACTTCTTTACATTCCAATACATACTTCTT<br>TACATTCCATTCACGCGTGGTACCCTCGAGGCCACCATGGCTACGTAACGTT<br>ATACGATGTCGCAGAGTATGCCGGTGTCTCTTATCAGACCGTTTCCCGCGTGG<br>TGAACCAGGCCAGCCACGTTTCTGCGAAAACGCGGGAAAAAGTGGAAGCGG

CGATGGCGGAGCTGAATTACATTCCCAACCGCGTGGCACAACAACTGGCGGG
CAAACAGTCGTTGCTGATTGGCGTTGCCACCTCCAGTCTGGCCCTGCACGCG
CCGTCGCAAATTGTCGCGGCGATTAAATCTCGCGCCGATCAACTGGGTGCCA
GCGTGGTGGTGTCGATGGTAGAACGAAGCGGCGTCGAAGCCTGTAAAGCGG
CGGTGCACAATCTTCTCGCGCAACGCGTCAGTGGGCTGATCATTAACTATCCG
CTGGATGACCAGGATGCCATTGCTGTGGAAGCTGCCTGCACTAATGTTCCGG
CGTTATTTCTTGATGTCTCTGACCAGACACCCATCAACAGTATTATTTTCTCCCA
TGAAGACGGTACGCGACTGGGCGTGGAGCATCTGGTCGCATTGGGTCACCA
GCAAATCGCGCTGTTAGCGGGCCCATTAAGTTCTGTCTCGGCGCGTCTGCGT
CTGGCTGGCTGGCATAAATATCTCACTCGCAATCAAATTCAGCCGATAGCGGA
ACGGGAAGGCGACTGGAGTGCCATGTCCGGTTTTCAACAAACCATGCAAATG
CTGAATGAGGGCATCGTTCCCACTGCGATGCTGGTTGCCAACGATCAGATGG
CGCTGGGCGCAATGCGCGCCATTACCGAGTCCGGGCTGCGCGTTGGTGCGG
ATATCTCGGTAGTGGGATACGACGATACCGAAGACAGCTCATGTTATATCCCGC
CGTTAACCACCATCAAACAGGATTTTCGCCTGCTGGGGCAAACCAGCGTGGA
CCGCTTGCTGCAACTCTCTCAGGGCCAGGCGGTGAAGGGCAATCAGCTGTTG
CCCGTCTCACTGGTGAAAAGAAAAACCACCCTGGCGCCCAATACGCAAACCG
CCTCTCCCCGCGCGTTGGCCGATTCATTAATGCAGCTGGCACGACAGGTTTC
CCGACTGGAAAGCGGGCAGGAATTCCCTAAGAAGAAGAGGAAGGTTGGGAG
CGGCGCCACAAACTTTTCCCTCCTGAAGCAGGCTGGAGATGTGGAGGAGAAT
CCCGGACCTAGCGGAATGTACGTGAGATTTGAGGTTCCTGAGGACATGCAGA
ACGAAGCTCTGAGTCTGCTGGAGAAGGTTAGGGAGAGCGGTAAGGTAAAGAA
AGGTACCAACGAGACGACAAAGGCTGTGGAGAGGGGACTGGCAAAGCTCGT
TTACATCGCAGAGGATGTTGACCCGCCTGAGATCGTTGCTCATCTGCCCCTCC
TCTGCGAGGAGAAGAATGTGCCGTACATTTACGTTAAAAGCAAGAACGACCTT
GGAAGGGCTGTGGGCATTGAGGTGCCATGCGCTTCGGCAGCGATAATCAACG
AGGGAGAGCTGAGAAAGGAGCTTGGAAGCCTTGTGGAGAAGATTAAAGGCCT
TCAGAAGAGATCTCATATGCATCTCGAGTACCCATACGATGTTCCAGATTACGC
TTGATCTAGAGTCGAATACATACTTCTTTACATTCCATCGACGAATTCACGCGAT
ACATACTTCTTTACATTCCAATACATACTTCTTTACATTCCA

LacO2-Kt2-**P21**-3xFlag

AATTGTGAGCGCTCACAATTCCCACAGTCGACCCTAGGTTGTGGAATTGTGAG
CGCTCACAATTGGGCGTGATCCGAAAGGTGACCCGAATTGGGCGTGATCCGA
AAGGTGACCCTAGTCCAGTGTGGTGGAATTC**ATGTCAGAACCGGCTGGGGAT**
GTCCGTCAGAACCCATGCGGCAGCAAGGCCTGCCGCCGCCTCTTCGGCCC
AGTGGACAGCGAGCAGCTGAGCCGCGACTGTGATGCGCTAATGGCGGGCT
GCATCCAGGAGGCCCGTGAGCGATGGAACTTCGACTTTGTCACCGAGACAC
CACTGGAGGGTGACTTCGCCTGGGAGCGTGTGCGGGGCCTTGGCCTGCCC
AAGCTCTACCTTCCCACGGGGCCCCGGCGAGGCCGGGATGAGTTGGGAGG
AGGCAGGCGGCCTGGCACCTCACCTGCTCTGCTGCAGGGGACAGCAGAGG
AAGACCATGTGGACCTGTCACTGTCTTGTACCCTTGTGCCTCGCTCAGGGGA
GCAGGCTGAAGGGTCCCCAGGTGGACCTGGAGACTCTCAGGGTCGAAAAC
GGCGGCAGACCAGCATGACAGATTTCTACCACTCCAAACGCCGGCTGATCT

**TCTCCAAGAGGAAGCCC**CTCGAGCTCGACGACTACAAAGACCATGACGGTG<br>ATTATAAAGATCATGACATCGACTACAAGGATGACGATGACAAGTAA
LacO2-Kt2-**Bax**-3xFlag
AATTGTGAGCGCTCACAATTCCCACAGTCGACCCTAGGTTGTGGAATTGTGAG<br>CGCTCACAATTGGGCGTGATCCGAAAGGTGACCCGAATTGGGCGTGATCCGA<br>AAGGTGACCCTAGTCCAGTGTGGTGGAATTCTCTAGAGCCACC**ATGGACGGG<br>TCCGGGGAGCAGCCCAGAGGCGGGGGGCCCACCAGCTCTGAGCAGATCAT<br>GAAGACAGGGGCCCTTTTGCTTCAGGGTTTCATCCAGGATCGAGCAGGGCG<br>AATGGGGGGGGAGGCACCCGAGCTGGCCCTGGACCCGGTGCCTCAGGATG<br>CGTCCACCAAGAAGCTGAGCGAGTGTCTCAAGCGCATCGGGGACGAACTG<br>GACAGTAACATGGAGCTGCAGAGGATGATTGCCGCCGTGGACACAGACTCC<br>CCCCGAGAGGTCTTTTTCCGAGTGGCAGCTGACATGTTTTCTGACGGCAACT<br>TCAACTGGGGCCGGGTTGTCGCCCTTTTCTACTTTGCCAGCAAACTGGTGCT<br>CAAGGCCCTGTGCACCAAGGTGCCGGAACTGATCAGAACCATCATGGGCTG<br>GACATTGGACTTCCTCCGGGAGCGGCTGTTGGGCTGGATCCAAGACCAGGG<br>TGGTTGGGGGCTGCCCCTGGCCGAGTCACTGAAGCGACTGATGTCCCTGTC<br>TCCAGGACGGCCTCCTCTCCTACTTTGGGACGCCCACGTGGCAGACCGTGA<br>CCATCTTTGTGGCGGGAGTGCTCACCGCCTCACTCACCATCTGGAAGAAGAT<br>GGGCTGAGGCCCCCAGCTGCCTTGGACTGTGTTTTTCCTCCA**CTCGAGCTC<br>GACGACTACAAAGACCATGACGGTGATTATAAAGATCATGACATCGACTACAAG<br>GATGACGATGACAAGTAA

Supplementary Table 2: the sequences of miRNA mimics used in this study

Name	Sequence (5' -> 3')
miR-21 mimics	UAGCUUAUCAGACUGAUGUUGA
miR-9 mimics	UCUUUGGUUAUCUAGCUGUAUGA
miR-124 mimics	UAAGGCACGCGGUGAAUGCC
miR-1 mimics	UGGAAUGUAAAGAAGUAUGUAU
